# The role of microRNA-325-3p as a critical player in cell death in NSCs and astrocytes 

**DOI:** 10.3389/fcell.2023.1223987

**Published:** 2024-01-16

**Authors:** Yukyeong Lee, Seung-Won Lee, Dahee Jeong, Hye Jeong Lee, Kinarm Ko

**Affiliations:** ^1^ Department of Stem Cell Biology, Konkuk University School of Medicine, Seoul, Republic of Korea; ^2^ Center for Genomic Medicine, Massachusetts General Hospital, Boston, MA, United States; ^3^ Department of Neurology, Harvard Medical School, Boston, MA, United States; ^4^ Cancer Early Detection Advanced Research Center, Knight Cancer Institute, Oregon Health & Science University, Portland, OR, United States; ^5^ Center for Stem Cell Research, Institute of Advanced Biomedical Science, Konkuk University, Seoul, Republic of Korea

**Keywords:** microRNA, neural stem cells, astrocytes, proliferation, differentiation, apoptosis

## Abstract

Neural stem cells (NSCs) are defined by their ability to self-renew and generate various cell types within the nervous system. Understanding the underlying mechanism by which NSCs proliferate and differentiate is crucial for the efficient modulation of *in vivo* neurogenesis. MicroRNAs are small non-coding RNAs controlling gene expression concerned in post-transcriptional control by blocking messenger RNA (mRNA) translation or degrading mRNA. MicroRNAs play a role as modulators by matching target mRNAs. Recent studies have discussed the biological mechanism of microRNA regulation in neurogenesis. To investigate the role of microRNAs in NSCs and NSC-derived glial cells, we screened out NSC-specific microRNAs by using miRNome-wide screening. Then, we induced downregulation by the sponge against the specific microRNA to evaluate the functional role of the microRNA in proliferation, differentiation, and apoptosis in NSCs and NSC-derived astrocytes. We found that microRNA-325-3p is highly expressed in NSCs and astrocytes. Furthermore, we showed that microRNA-325-3p is a regulator of apoptosis by targeting brain-specific angiogenesis inhibitor (BAI1), which is a receptor for apoptotic cells and expressed in the brain and cultured astrocytes. Downregulation of microRNA-325-3p using an inducible sponge system induced cell death by regulating BAI1 in NSCs and NSC-derived astrocytes. Overall, our findings can provide an insight into the potential roles of NSC-specific microRNAs in brain neurogenesis and suggest the possible usage of the microRNAs as biomarkers of neurodegenerative disease.

## 1 Introduction

MicroRNAs are a class of conserved non-coding RNAs containing about 22 nucleotides that modulate gene expression by targeting messenger RNA (mRNA), which leads to reduced translational efficiency, thereby influencing many biological processes. Large numbers of microRNAs contribute to neural maturation processes and have been found to have distinct expression patterns. Furthermore, many microRNAs are dynamically regulated during central nervous system (CNS) development and are spatially expressed in the adult brain, indicating their essential roles in neural development and function (Rajasethupathy et al., 2009; Cao and Chan, 2016). In addition, the dysfunction of microRNAs contributes to neurological diseases (Cao and Chan, 2016). MicroRNAs are a class of small non-coding RNAs that have been shown to play key roles in neural development and stem cell proliferation and differentiation (Bonev et al., 2011; Coolen et al., 2013; Radhakrishnan and Anand, 2016). MicroRNA-9, which is known to be most highly expressed in neural development and the adult vertebrate brain, has been extensively studied for its association with neurodegenerative diseases such as Huntington’s, Alzheimer’s, and Parkinson’s diseases (Kim et al., 2007; Cogswell et al., 2008; Hébert et al., 2008; Packer et al., 2008; Buckley et al., 2010; Coolen et al., 2013; Zhang et al., 2013; Shaik et al., 2018).

Neural stem cells (NSCs) are a subset of undifferentiated precursor cells that can self-renew and have the capacity to give rise to neuronal and glial lineages (Gage, 2000). The process of functions including NSC proliferation, migration, and fate specification are regulated by the dynamic interplay between microRNA regulators and cell-extrinsic signals (Shi et al., 2008; Zhao et al., 2010; Wang et al., 2013). Abnormal microRNA expression due to overexpression or inhibition may interfere with a general mechanism between NSC proliferation and differentiation (Zhao et al., 2009; Zhao et al., 2010; Wang et al., 2013; Zhang et al., 2015; Miao et al., 2017). In the CNS, glial cells significantly influence cell structure similar to cell homeostasis. Astrocytes, also known collectively as astroglia, participate in all essential CNS functions including homeostasis and neurogenesis (Aschner, 1998; Becher et al., 2000; Simard and Nedergaard, 2004; Otaegi et al., 2011; Oberheim et al., 2012; Howarth, 2014; Tay et al., 2015). Previous studies suggest that microRNAs can directly modify the glial phenotype and cause astrocyte dysfunction and promote astrocyte proliferation in models of chronic spinal cord injury (Hoye et al., 2018; Wang et al., 2018). Research on specific microRNA expression involving NSCs has been conducted, however, studies on the role of specifically enriched microRNAs in NSCs and astrocytes are still scarce.

In this study, we aimed to find microRNAs highly expressed in NSCs and astrocytes by miRNome-wide screening based on quantitative reverse transcription polymerase chain reaction (qRT-PCR). Furthermore, we investigated the role of highly expressed microRNAs in NSCs and astrocytes in cell proliferation and differentiation and its target gene.

## 2 Materials and methods

### 2.1 NSC culture

The mouse NSCs used in this study were originally generated in our previous study (Lee et al., 2015). NSCs were cultured on Matrigel (Corning)-coated 24-well plates and under expansion conditions, with the culture medium consisting of DMEM/F12 (Corning) with N2 supplement (Gibco, United States), B27 supplement without vitamin (Gibco), penicillin/streptomycin (Gibco), non-essential amino acids (Gibco), β-mercaptoethanol (Gibco), fibroblast growth factor-basic (PeproTech, Korea, 20 ng/mL), and platelet-derived growth factor-AA (Prospec, East United States, 20 ng/mL).

### 2.2 Differentiation into astrocytes and neurons

For differentiation into astrocytes, NSCs were seeded at 2.5 × 10^5^ cells on Matrigel-coated 24-well plates. The following day, the cells were fed with astrocyte differentiation medium, consisting of DMEM/F12 with 15% fetal bovine serum (FBS; Corning, United States), N2 supplement (Gibco, United States), penicillin/streptomycin (Gibco), non-essential amino acids (Gibco), and β-mercaptoethanol (Gibco) for 7 days. For the differentiation of NSCs into neurons, NSCs were seeded at 1.5 × 10^5^ cells on poly-l-ornithine/laminin–coated 24-well plates. During the first 4 days, the cells were cultured in the neuronal differentiation medium containing DMFM/F12 (Corning), B27 supplement (Gibco), N2 supplement (Gibco), penicillin/streptomycin (Gibco), non-essential amino acids (Gibco), β-mercaptoethanol (Gibco), and fibroblast growth factor-basic (20 ng/mL; PeproTech). On day 4 of neuronal differentiation, the medium was replaced without fibroblast growth factor-basic, and the cells were cultured until day 8 of neuronal differentiation.

### 2.3 MicroRNA expression analysis by MicroRNA profiling

The quantitative PCR (qRT-PCR) array was used for parallel quantitative analysis of microRNA expression. MicroRNA profiling was performed using the Mouse miRNome Sanger miRBase microRNA Profiler Set (#RA670A-1, System Biosciences, United States). The expression of mouse microRNAs (based on Sanger miRBase version 14) was analyzed as per the manufacturer’s instructions. https://www.systembio.com/wp/wp-content/uploads/2020/10/miRNome_Profilers_Manual_WEB-1.pdf. The microRNA array data have been deposited in the Gene Expression Omnibus (GEO) under the accession number GSE241920 (https://www.ncbi.nlm.nih.gov/geo/query/acc.cgi?acc=GSE241920).

### 2.4 Differentially expressed microRNA analysis

MicroRNA microarray data were normalized by logarithmic transformation, and values not measured in the microarray were removed. MicroRNA expression difference values between MEF, ESC, and NSC were visualized using the pheatmap R package.

### 2.5 Construction of MicroRNA sponge

Lentiviral green fluorescent protein (GFP)-microRNA sponge directed against the microRNA-325-3p vector was designed based on the pLVTHM (#12247, Addgene, United States) plasmid. First, the microRNA sponge was inserted by using T4 ligase into the Bspd1 (NEB, United States)- and Mlu1 (NEB)-digested pLVTHM plasmid and transformed into DH5α by using the heat shock method. Furthermore, selected colonies were incubated in Luria–Bertani broth with ampicillin for minipreparation. For self-ligation, the plasmid was digested with the Bspd1 and Mlu1 restriction enzyme and electroporation was performed to confirm the size.

To insert the plasmid inserted with the microRNA sponge into the inducible system vector pLVET-tTR-KRAB (#11644, Addgene), the backbone and insert were digested with the restriction enzymes Fsp1 and Msc1, and the cut insert was ligated into the backbone plasmid to generate an inducible microRNA sponge (Cosmogenetech, South Korea).

### 2.6 Lentivirus production

Lentiviruses were produced in HEK293T (293T) packaging cells in culture medium with DMEM containing 10% FBS by effect vector, packaging vector ps-PAX2 (#12260, Addgene), and envelop vector VSV-G (#12259, Addgene) for 48 h. The supernatant was transferred to a 50-mL conical tube and mixed with a Lenti-X Concentrator (Clontech, United States). After overnight incubation at 4°C, viral particles were collected and suspended in a conditioned medium.

### 2.7 Cell transduction

Viral particles suspended in the NSC culture medium were added to 1 × 10^4^ NSCs on a Matrigel-coated 12-well plate with polybrene (5 μm/mL; Sigma Aldrich) for 48 h. After 2 days, the medium was carefully removed by using a pipette, and fresh NSC medium was added.

### 2.8 Fluorescence-activated cell sorting (FACS)

After lentivirus infection to NSCs, the infected cells were harvested and filtered using a 70-μm cell strainer. The filtered cells were washed with Dulbecco’s phosphate-buffered saline (DPBS) and transferred into a new FACS tube. An FACS Aria I flow cytometer (BD Biosciences, United States) was used by an operator to separate and sort GFP-positive cells.

### 2.9 Cell counting

Cell counting was conducted for every culture using an automated cell counter (Bio-Rad, United States) and counter slides (Bio-Rad) with trypan blue (Sigma Aldrich, United States). The numbers in the graphs were conceived by carefully checking them three times.

### 2.10 3-(4,5-Dimethylthiazol-2-yl)-2,5-diphenyltetrazolium bromide (MTT) assay

Fresh NSC medium 900 μL and MTT solution 100 μL (Sigma) were added into each well of a 96-well plate, and the plate was incubated at 37°C for 3 h. After incubation, the MTT solution was replaced with DMSO (Sigma, United States) and incubated on an orbital shaker for 15 min. The absorbance of the plate was read at OD = 540 nm.

### 2.11 Annexin-V assay

The cells were collected by centrifugation and resuspended in 500 μL of binding buffer and 5 μL of annexin v-Cy3 (BioVision, United States) and incubated at room temperature (RT) for 10 min in the dark. The stained cells were analyzed by flow cytometry using an FL2 channel for annexin v-cy3.

### 2.12 Propidium iodide (PI) assay

The cells were stained with propidium iodide (PI) (Sigma-Aldrich) in binding buffer and incubated at RT for 15 min in the dark. After incubation, the stained cells were analyzed by flow cytometry.

### 2.13 Total RNA and small RNA extraction

To extract total RNA or microRNA-enriched RNA, we used an RNeasy and miRNeasy Mini Kit (Qiagen, Germany), following the supplier’s instructions.

### 2.14 cDNA synthesis

Total RNA (1 μg) was reverse-transcribed into cDNA using a high-capacity cDNA reverse transcription kit (Applied Biosystems, United States) and miScript RT (Qiagen) following the manufacturer’s protocol.

### 2.15 PCR

All reverse transcription polymerase chain reactions (RT-PCRs) used Ex Taq polymerase (TaKaRa, Japan) and were performed for 25–28 cycles for all markers. The primers used to amplify cDNA samples are listed in Supplementary Table S1. Gene expression levels were evaluated by qRT-PCR using a Roche real-time PCR system (Roche, Switzerland) and SYBR Green (Thermo Scientific, United States). Primer sequences are listed in Supplementary Table S1.

### 2.16 Immunocytochemistry

The cells were fixed in 4% paraformaldehyde for 10 min at RT and washed with Dulbecco’s phosphate-buffered saline (DPBS; HyClone, United States). For cell permeabilization, 0.5% Triton X-100 (Sigma-Aldrich) in DPBS was added to the fixed cells for 10 min at RT, and the cells were blocked with 2% of bovine serum albumin (Gibco) in DPBS for 1 h at RT. Then, the cells were rinsed and incubated in the primary antibody solution overnight at 4°C and washed with 0.2% Tween 20 in DPBS. After washing, the cells were incubated in the secondary antibody solution for 1 h at RT and washed with 0.2% Tween 20 in DPBS. For nuclear acid staining, the cells were incubated in the 4′-6-diamidino-2-phenylindole (DAPI) antibody for 5 min at RT and washed with 0.2% Tween 20 in DPBS. Antibody information is shown in Supplementary Table S2.

### 2.17 Western blotting

The cells were lysed in RIPA lysis buffer (Thermo Scientific) with a protease inhibitor cocktail (Thermo Scientific). The samples were run on 8% SDS-PAGE gel for 20 min at 80 V and 40 min at 120 V and then transferred to a transfer membrane (Pall Life Sciences, United States) for 90 min at 110 V. The membrane was blocked in 5% skimmed milk in Tris-buffered saline with Tween 20 (TBST) for 1 h at RT. The membrane was incubated with the primary antibody in blot buffer overnight at 4°C and washed three times for 5 min each with TBST. Then, the blots were incubated with a secondary antibody conjugate to HRP for 1 h at RT. The membrane was washed three times for 5 min each with TBST and incubated with an appropriate luminol-based chemiluminescent substrate. After incubation, the membrane was visualized on an ImageQuant LAS 4000. Images were quantified using ImageStation and ImageJ software. Antibody information is shown in Supplementary Table S3.

### 2.18 Gene prediction

To search for mRNA that might regulate mmu-miR-325-3p expression, we used the following public prediction algorithms and databases (miRDB and MGI):

https://www.informatics.jax.org/interaction/explorer?markerIDs=MGI:3619336 and https://mirdb.org/cgi-bin/search.cgi?searchType=miRNA&full=mirbase&searchBox=MIMAT0000558.

### 2.19 Statistical analysis

All data are presented as means ± SEM. All statistics were calculated using independent *t*-tests or analysis of variance (ANOVA) with the least significant difference test for *post hoc* analysis. A value of *p* < 0.05 was considered significant.

## 3 Results

### 3.1 Identification of NSC-specific microRNA by miRNome-wide screening and qRT-PCR

**FIGURE 1:**
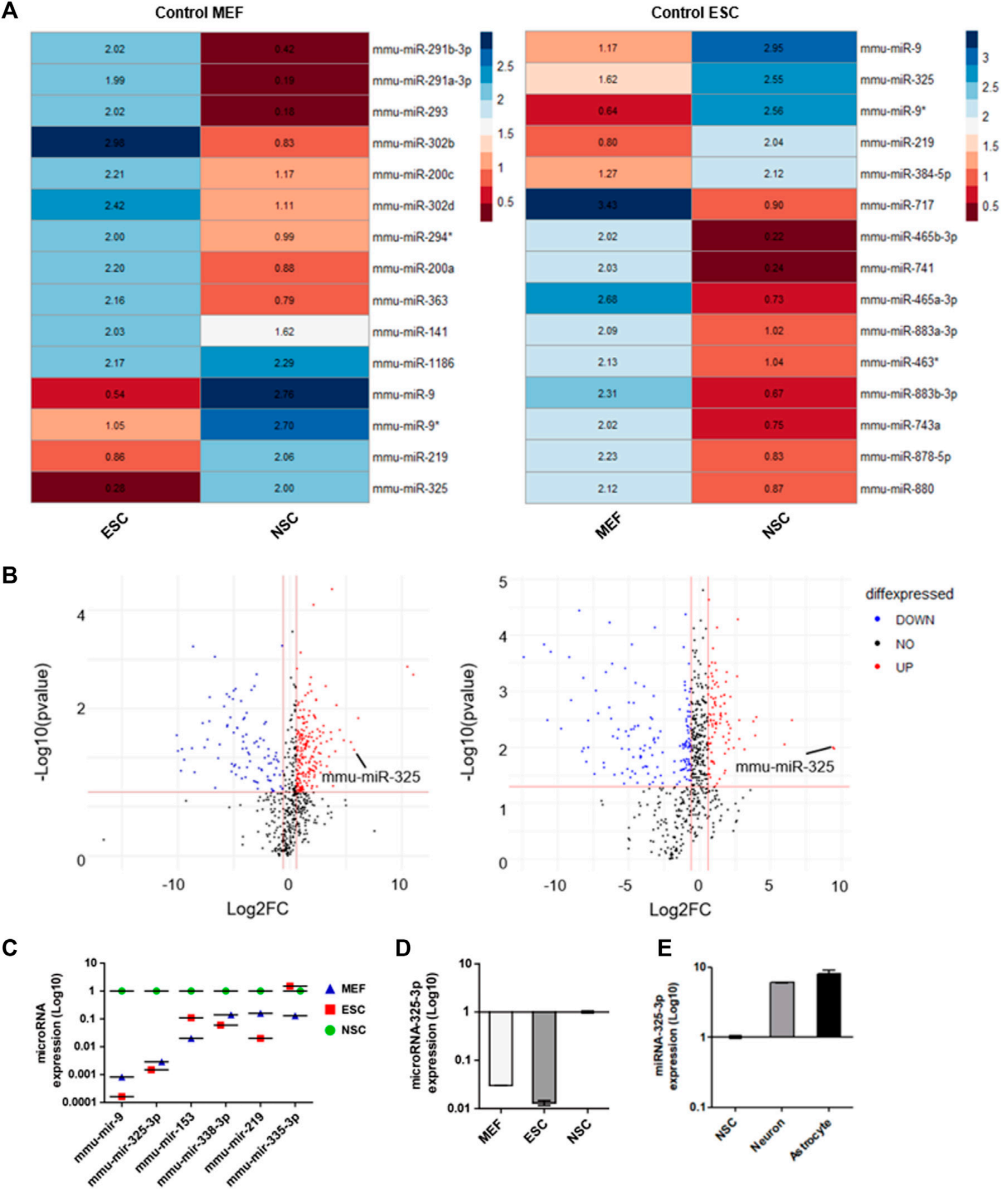
Identification of NSC-specific microRNA by miRNome-wide screening and qRT-PCR. **(A)** Heatmap analysis of miRNome expression profiles of MEFs, ESCs, and NSCs. Heatmaps were generated with 50-fold higher expression. R was used for analysis. **(B)** Volcano plot. The log2 FC indicates the expression level for each microRNA after the normalization to MEFs (left) and ESCs (right). **(C)** Representative dot graph based upon microRNA expression levels in MEFs, ESCs, and NSCs. **(D)** qRT-PCR analysis. Representative bar graph based upon microRNA-325-3p expression levels in MEFs, ESCs, and NSCs. **(E)** qRT-PCR analysis. Representative bar graph based upon microRNA-325-3p expression levels in NSCs, neurons, and astrocytes. Graphs represent changes after normalization to NSCs. Data are presented as the means ± SEM (*n* = 3).

The samples consisting of MEFs, ESCs, and NSCs were used to identify NSC-specific microRNA. Small non-coding RNA was extracted from these samples. Then, we screened out NSC-specific microRNAs by miRNome-wide screening and confirmed by using real-time PCR. Heatmap analysis data indicated candidates that had a relatively high number of NSCs compared to MEFs and ESCs (Figure 1A). After normalization to the MEF group, microRNAs highly expressed in NSCs were identified (Figure 1A; left panel). Similarly, after normalization to the ESC group, microRNAs highly expressed in NSCs were identified (Figure 1A; right panel).

Based on these results, it was confirmed that microRNA-325 was highly expressed specifically in NSCs. Following the heatmap analysis data, we profiled microRNAs that were expressed highly in NSCs (Figure 1B and Supplementary Figure S1) by using a volcano plot. Among these microRNAs, we found microRNA-325-3p, which had the highest expression in NSCs, followed by brain- and neuronal-specific microRNA-9, which is known to play an important role in neuronal development (Figure 1C). Furthermore, we confirmed through qRT-PCR analysis that NSC-specific microRNA is highly expressed in NSCs (Figure 1D). In addition, we observed an increase in the expression of microRNA-325-3P in NSC-derived neurons and astrocytes after induced differentiation, suggesting that microRNA-325-3p is not only NSC-specific but also could play a critical role in the differentiation into the neuronal or glial lineage (Figure 1E).

### 3.2 Establishment of GFP-lentiviral microRNA sponge plasmid and GFP-positive infected cells

**FIGURE 2:**
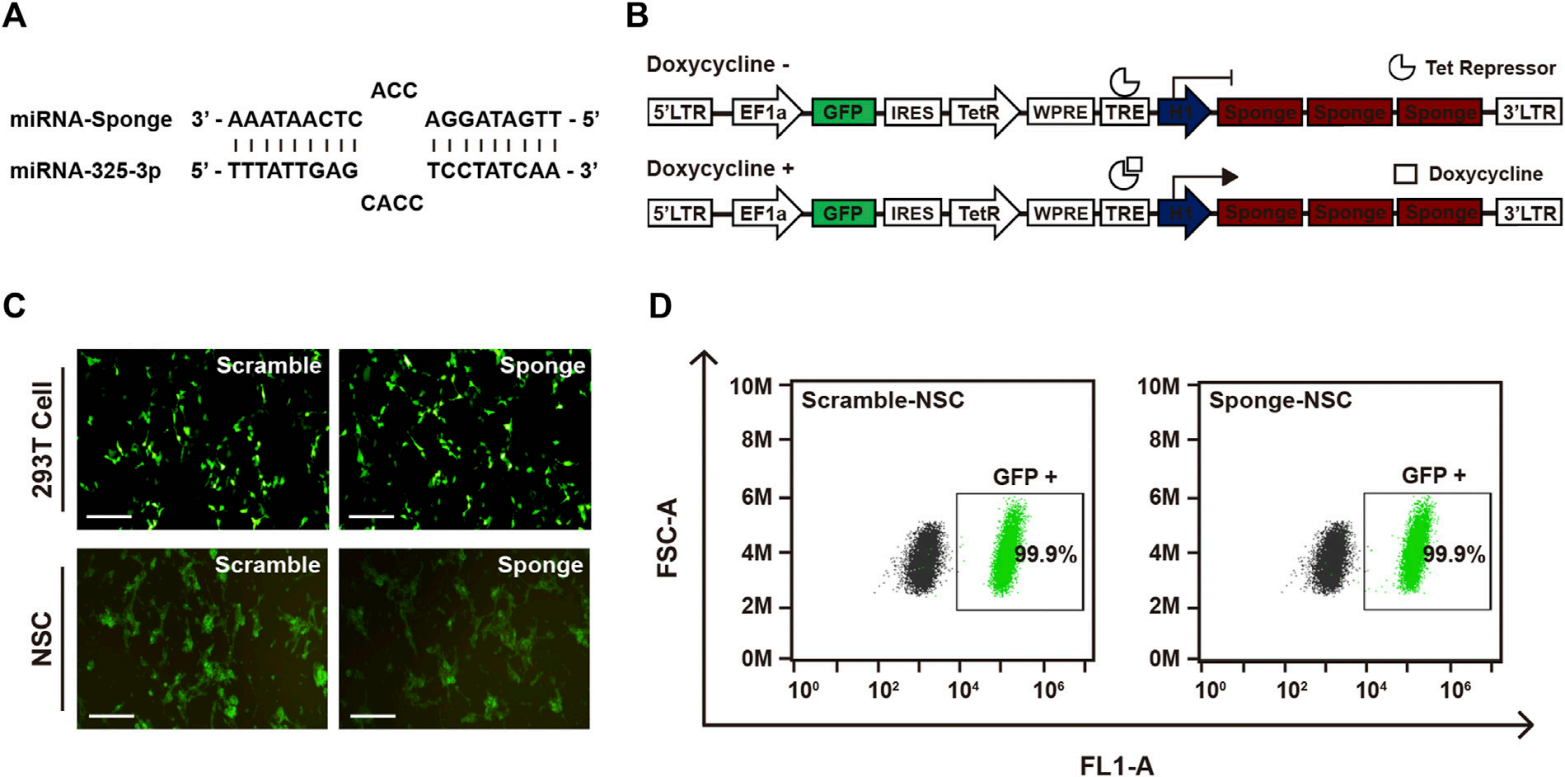
Establishment of the GFP-lentiviral microRNA sponge plasmid vector. **(A)** MicroRNA-325-3p binding site of a microRNA sponge. **(B)** Schematic representation of the inducible system and mechanism. In the absence of doxycycline or tetracycline, microRNA sponge expression is repressed by Tet repressor protein. MicroRNA sponge can be expressed by adding doxycycline or tetracycline. **(C)** Representative fluorescence microscopy images of 293T cells transfected using the scramble plasmid vector and microRNA-325-3p sponge plasmid vector and images of NSCs after lentiviral infection using the scramble plasmid vector and microRNA-325-3p-sponge plasmid vector. **(D)** Representative flow cytometry analysis of the population of negative cells (mock NSCs) and infected cells (scramble NSCs and sponge NSCs). Scale bars: 250 μm.

To investigate the role of microRNA-325-3p in NSCs and NSC-derived differentiated cells, we designed a microRNA-325-3p-sponge carrying anti-sequence for binding to the microRNA-325-3p (Figure 2A). For generating an inducible system of sponge expression, we inserted the microRNA-325-3p-sponge into the EF1α/EGFP/TetR plasmid. To enhance the binding efficiency, three sites were designed on the sponge for binding to the target microRNA (Figure 2B). After generating lentiviral particles, the viral supernatant was concentrated with a concentrator and added to NSCs for 48 h to establish lentiviral-infected cells. For accurate separation of lentiviral-infected cells, only GFP-positive cells were cultured after fluorescence-activated cell sorting (Figure 2C). The GFP expression of the cultured cells was confirmed by flow cytometry (Figure 2D), and viral integration of the infected cells was confirmed by genomic DNA sequencing (Supplementary Figure S2).

To confirm the properties of NSCs in the infected group, we examined the mRNA and protein expression of NSC-specific genes in the infected NSCs (Supplementary Figures S3A, B). Additionally, we validated the differentiation ability of the infected NSCs by demonstrating their capacity to differentiate into neurons and astrocytes (Supplementary Figure S3C). These data showed that the infected cells had the characteristics of NSCs. Therefore, we successfully established a cell line capable of modulating the expression of microRNA-325-3p, a regulator of cell proliferation and differentiation in NSCs, by using an inducible system with the microRNA sponge.

### 3.3 The role of NSC-specific microRNA-325-3p in proliferation and apoptosis in NSCs

**FIGURE 3:**
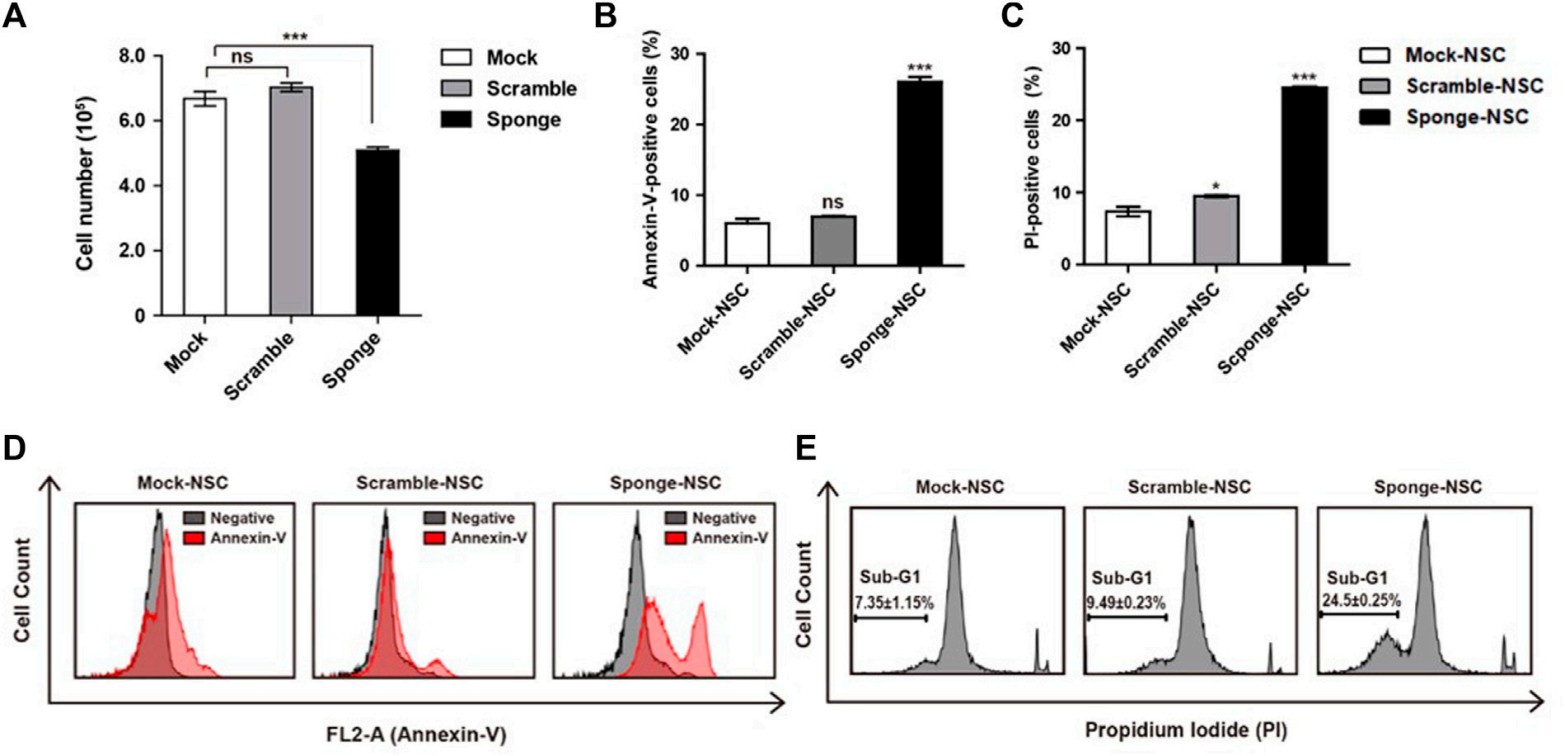
Role of NSC-specific microRNA-325-3p in proliferation and apoptosis in NSCs. **(A)** Representative bar graphs based upon the counting of the cell numbers in mock NSCs, scramble NSCs, and sponge NSCs after the induction of microRNA-325-3p downregulation using trypan blue staining. **(B)** Representative bar graphs based upon the annexin-v-positive cell population. **(C)** Representative bar graphs based on the PI-positive cell population. **(D)** Flow cytometry analysis of apoptosis in mock NSCs, scramble NSCs, and sponge NSCs by annexin-v staining after the induction of microRNA-325-3p downregulation using a sponge. **(E)** Flow cytometry analysis of apoptosis in mock NSCs, scramble NSCs, and sponge NSCs by PI staining after the induction of microRNA-325-3p downregulation using a sponge. Data are presented as means ± SEM (*n* = 3). *p < 0.05, ***p < 0.001.

MicroRNAs are known to be important to cell viability and proliferation. Here, we investigated a downregulation of microRNA-325-3p that affects the proliferation of NSCs by carrying out trypan blue and MTT assays. To induce downregulation of microRNA-325-3p with an inducible doxycycline system, doxycycline (final concentration: 100 ng/mL; Sigma-Aldrich) was added into the NSC culture medium for 72 h. The data showed that there was no difference between mock NSCs and scramble NSCs, but there was a small but significant decrease in the number of cells in sponge NSCs when downregulation of microRNA-325-3p was induced by the sponge (Figure 3A). Consistently, in the MTT assay, cell viability tended to decrease in sponge NSCs (Supplementary Figure S4). In addition, an annexin-v assay was performed with flow cytometry to measure the extent of apoptosis in cells (Aubry et al., 1999). The results show that the difference between control and staining treatment in mock NSCs and scramble NSCs was approximately 8%, while in sponge NSCs, labeled cells were increased to approximately 25% (Figures 3B, D). These results show that microRNA-325-3p downregulation can inhibit the proliferative capacity of NSCs and induce apoptosis.

To confirm cell death, an assay for the observation of the sub-G1 population was performed using propidium iodide (PI) staining in combination with flow cytometry. As shown in the results, in mock NSCs and scramble NSCs, there was no difference in the ratio of sub-G1 population of cells (Figures 3C, E). On the other hand, when the downregulation of microRNA-325-3p was induced by the sponge in sponge NSCs, the sub-G1 population of cells increased by approximately 15% compared with mock NSCs and scramble NSCs (Figures 3C, E). These results indicate that the downregulation of microRNA-325-3p induces accumulation in the sub-G1 phase population and that microRNA-325-3p regulates cell proliferation and apoptosis by targeting proliferation and apoptosis-related genes directly.

### 3.4 Apoptotic regulation of microRNA-325-3p in astrocytes from NSCs

**FIGURE 4:**
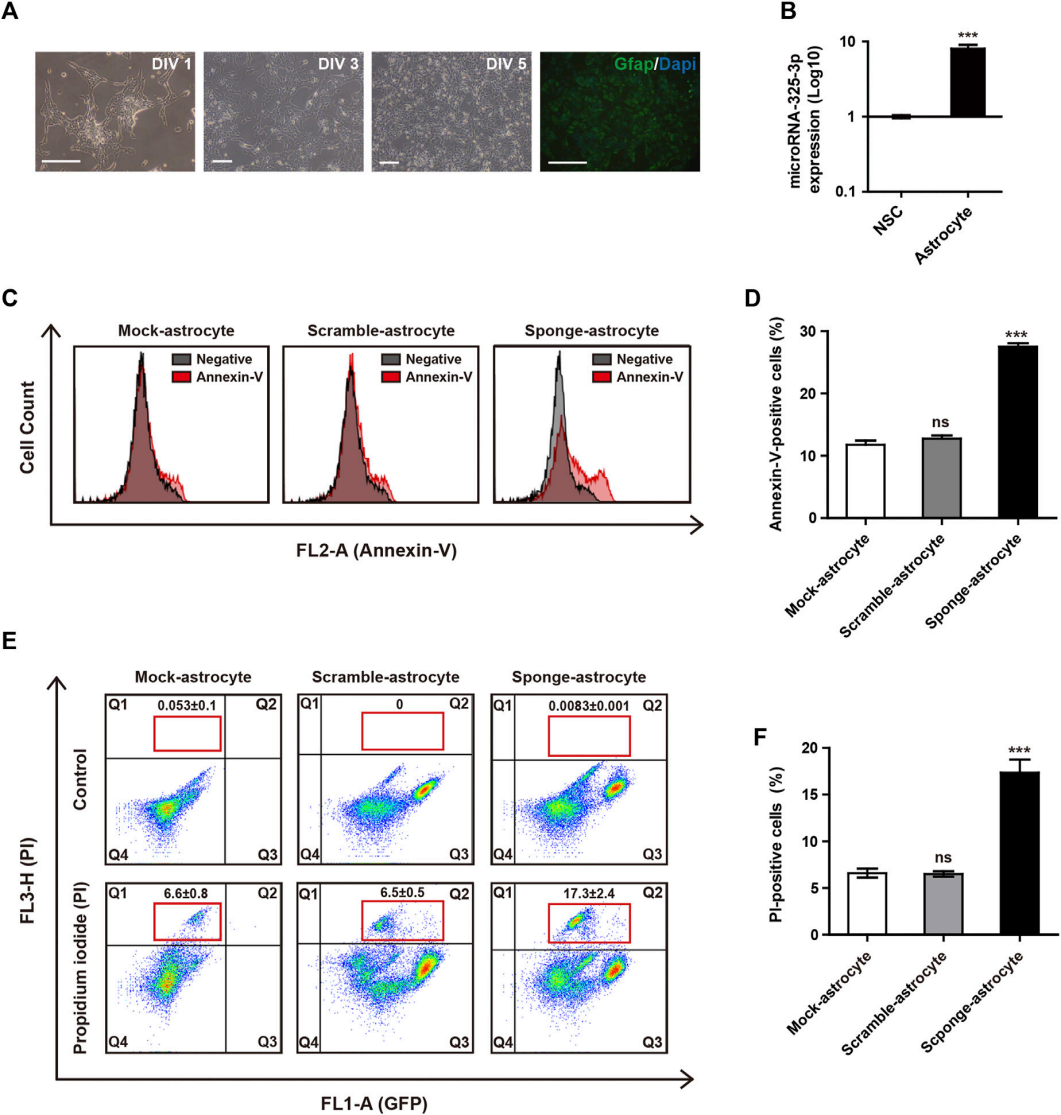
Apoptotic regulation of microRNA-325-3p in astrocytes from NSCs. **(A)** Representative images of differentiation into astrocytes at days *in vitro* (DIV)1, DIV 3, and DIV 5 and immunofluorescence images of GFAP-positive astrocytes at DIV5. **(B)** qRT-PCR analysis. Representative bar graph based upon microRNA-325-3p expression levels in NSCs and NSC-derived astrocytes. Graphs represent changes after normalization to NSCs. **(C)** Flow cytometry analysis of apoptosis in mock NSC-derived astrocytes, scramble NSC-derived astrocytes, and sponge NSC-derived astrocytes by annexin-v staining after the induction of microRNA-325-3p downregulation using a sponge. **(D)** Representative bar graphs based upon the annexin-v-positive cell population. **(E)** Flow cytometry analysis of apoptosis in mock NSC-derived astrocytes, scramble NSC-derived astrocytes, and sponge NSC-derived astrocytes by PI staining after the induction of microRNA-325-3p downregulation using a sponge. **(F)** Representative bar graphs based on the PI-positive cell population. Data are presented as means ± SEM (*n* = 3). ***p < 0.001. Scale bars: 250 μm.

By conducting qRT-PCR analysis, we identified that the expression of microRNA-325-3p increases after differentiation into astrocytes from NSCs (Figures 4A, B). These results suggest the potential of microRNA-325-3p as a regulatory factor involved in the generation and function of astrocytes. To analyze whether the downregulation of microRNA-325-3p by the sponge affects astrocyte cell death upon differentiation, we used annexin-v and PI staining to analyze the population of cell death. Annexin-v analysis shows an approximate 16% increase in the population undergoing apoptosis in sponge NSC-derived astrocytes compared to mock NSC-derived astrocytes and scramble NSC-derived astrocytes (Figures 4C, D). Consistently, the populations of PI-positive cells were 6.6% ± 0.8% in mock NSCs, 6.5% ± 0.5% in scramble NSCs, and 17.3% ± 2.4% in sponge NSCs (Figure 4E). The graph shows a 10.5% increase in PI-positive cells during astrocyte differentiation in the group that induced downregulation with sponge expression (Figure 4F). These results suggest that microRNA-325-3p is involved in the differentiation from NSCs to astrocytes and targets genes related to cell differentiation into astrocytes and cell death in astrocytes.

### 3.5 Role of microRNA-325-3p as a post-transcriptional regulator by targeting the apoptosis-related gene BAI1 in NSCs and NSC-derived astrocytes

**FIGURE 5:**
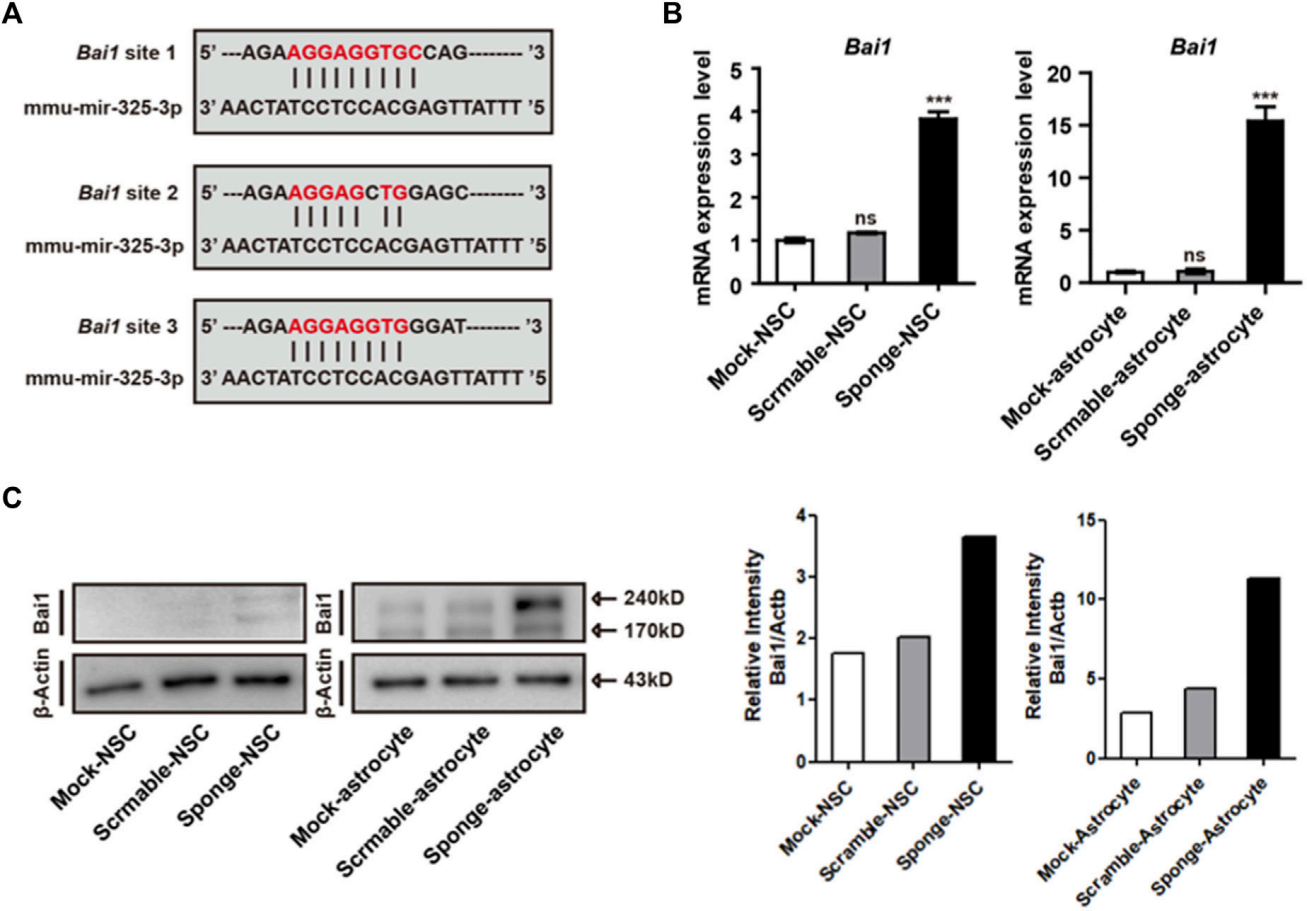
*Bai1* is the target of microRNA-325-3p in NSCs and NSC-derived astrocytes. **(A)** *Bai1* possesses binding sites for microRNA-325-3p in their 3′UTRs. **(B)** qRT-PCR analysis. Representative bar graphs based upon apoptosis-related gene *Bai1* mRNA expression levels in NSCs and NSC-derived astrocytes after the induction of microRNA-325-3p downregulation using a sponge. Graphs represent changes after normalization to mock NSC-derived astrocytes. **(C)** Representative images of Western blot analysis. The protein expression level of Bai1 (left) and relative intensity (right) in NSCs and NSC-derived astrocytes. β-Actin served as an internal control. Data are presented as means ± SEM (*n* = 3). ***p < 0.001.

Before the examination of differentiation and apoptosis-related regulation, we used prediction programs (miRDB and MGI) to search for the target of microRNA-325-3p, and candidate genes with the highest scores were screened out. Moreover, by using a database, we found that microRNA325-3p has binding sites in the 3’-untranslated region (UTR) of brain-specific angiogenesis inhibitor (*Bai1*) (Figure 5A). *Bai1* has a conversed stretch of seven to nine nucleotides that can base-pair with the corresponding microRNA-325-3p. Furthermore, the qRT-PCR analysis revealed a significant increase in the mRNA expression pattern of *Bai1* in NSC-derived astrocytes and a small increase in NSCs by microRNA-325-3p downregulation (Figure 5B). Furthermore, Western blot showed that microRNA-325-3p was a negative regulator of BAI1 in NSCs and NSC-derived astrocytes (Figure 5C). These results suggest that microRNA-325-3p targets and regulates BAI1 expression in NSCs and NSC-derived astrocytes.

## 4 Discussion

In this study, we found the NSC- and astrocyte-specific microRNA-325-3p by miRNome-wide screening. Based on the screening data by qRT-PCR, microRNA-325-3p shows high expression in NSCs and astrocytes. MicroRNA-325-3p has been reported to be downregulated in neurodegeneration and upregulated after brain damage (Barak et al., 2013; Yang et al., 2017). Our study found the role of microRNA-325-3p in NSCs and astrocytes, suggesting that microRNA-325-3p can play an important role in the nervous system or brain, like the existing CNS or brain-related microRNA studies. As a result of the microRNA sponge system, we identified that the downregulation of microRNA-325-3p induces apoptosis in NSCs. Furthermore, we confirmed that the expression of microRNA-325-3p increases after differentiation into astrocytes from NSCs, and the downregulation of microRNA-325-3p induces apoptosis in astrocytes (Figures 4B–F). Glial cells play a key role in maintaining homeostasis in the brain and CNS. Among these cells, astrocytes participate in all essential CNS functions (Oberheim et al., 2012; Maia et al., 2015; Phatnani and Maniatis, 2015). Thus, the regulation of astrocyte apoptosis is crucial in the physiology in the CNS. Previous studies have highlighted the role of microRNAs in regulating apoptosis in astrocytes and specific populations of astrocytes (Aw et al., 2017; Zheng et al., 2017). Additionally, microRNA-325-3p, identified as being downregulated in human hepatocellular carcinoma cells and tissues, modulates cell proliferation and apoptosis by directly targeting the tumor suppressor AQP5 (Zhang et al., 2019). This emphasizes the significant involvement of microRNAs in the regulation of cell death and apoptosis. Consistent with earlier findings, we speculate that microRNA-325-3p may play an important role in brain development and neurological diseases associated with astrocytes. These results suggest that microRNA-325-3p might be a modulator playing an important role in astrocytes as well as NSCs and imply that microRNA-325-3p plays a role in NSC and astrocyte apoptosis.

MicroRNAs control translational repression by blocking or degrading mRNA. In the prediction program to search for the target of microRNA-325-3p, we screened out candidate genes with the highest score. Then, we found that BAI1 has an association with cell death and glial cells and with the corresponding binding site with microRNA-325-3p. BAI1 was initially identified as a p53-inducible gene, specifically expressed in the brain, and expression of BAI1 has been demonstrated in neurons and astrocytes (Nishimori et al., 1997; Mori et al., 2002; Sokolowski et al., 2011). BAI1 was also known as a receptor for apoptotic cells and was proven to function in the engulfment of apoptotic cells (Park et al., 2007). BAI1-expressing astrocytes include engulfed apoptotic debris, and BAI1 expressed in cultured astrocytes showed accumulation within the phagocytic cup (Sokolowski et al., 2011). Furthermore, BAI1 is expressed by phagocytes and is found in phagosomes (Das et al., 2014). We found that microRNA-325-3p mediates the apoptosis of NSCs and NSC-derived astrocytes by directly targeting BAI1, which suggests the potential of microRNA-325-3p as a regulator of cell death and differentiation into glial cells in NSCs and NSC-derived astrocytes.

In conclusion, our study suggests microRNA-325-3p as a new critical player in the apoptosis of NSCs and astrocytes. Our work is a cornerstone in apoptosis research using microRNA-325-3p as a biomarker in the BAI1 expressed in the brain and CNS and might be a key to investigating the correlation between microRNA-325-3p and neurodegenerative disease associated with apoptosis.

## Data Availability

The datasets presented in this study can be found in online repositories. The names of the repository/repositories and accession number(s) can be found in the article/Supplementary Material.
