# Supplementary material for: The role of microRNA-325-3p as a critical player in cell death in NSCs and astrocytes
Source: Front Cell Dev Biol. 2024 Jan 16;11:1223987. doi: 10.3389/fcell.2023.1223987 (PMC10877600; doi:10.3389/fcell.2023.1223987)
Supplement: Supplementary file 3 [file Table1.DOCX]

Supplementary Material

**The Role of MicroRNA-325-3p**

**as a Critical Player in Cell Death in NSCs and Astrocytes**

Yukyeong Lee^1,3,4^, Seung-Won Lee^1,5^, Dahee Jeong^1^, Hye Jeong Lee^1^, Kinarm Ko^1,2*^

*** Correspondence:** Kinarm Ko: knko@kku.ac.kr

# Supplementary Figures and Tables

**1.1 Supplementary Figures**

**
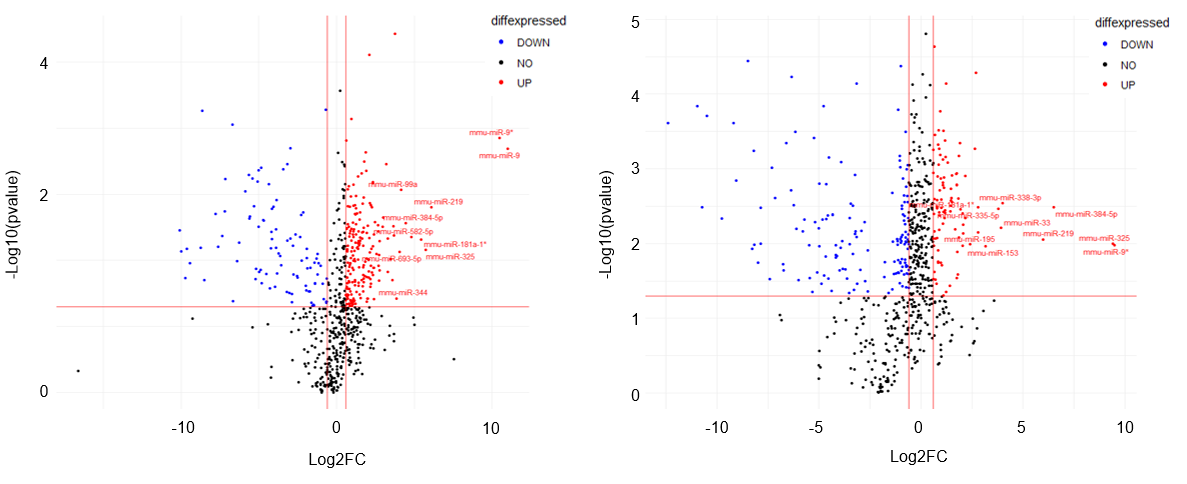
**

**Supplementary Figure 1.** Volcano plot. Top 10 highly expressed microRNA in NSCs. The log2 FC indicates the expression level for each microRNA after normalized to MEF (left) and ESC (right).

**
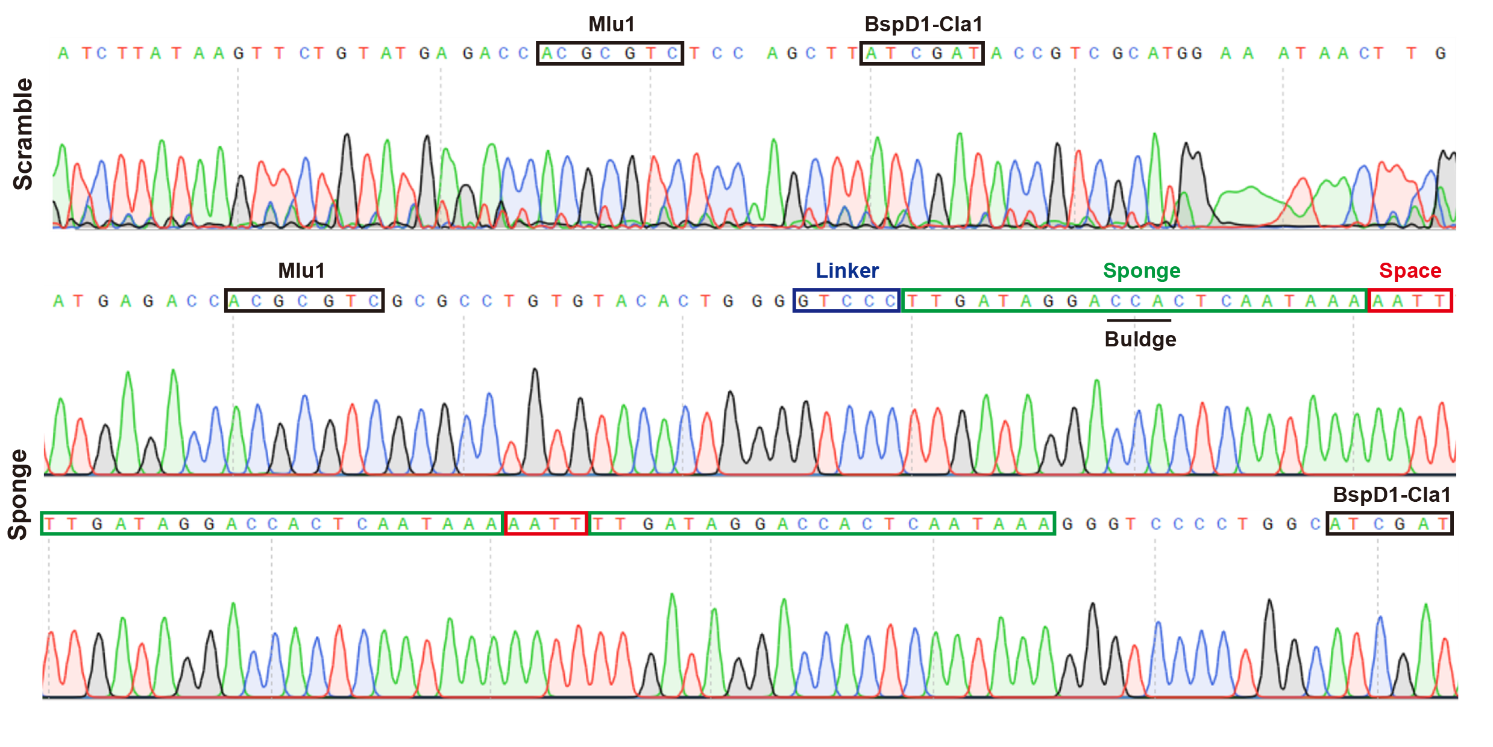
**

**Supplementary Figure 2.** DNA sequencing analysis of scramble NSCs and sponge NSCs that have anti sequence to microRNA-325-3p.


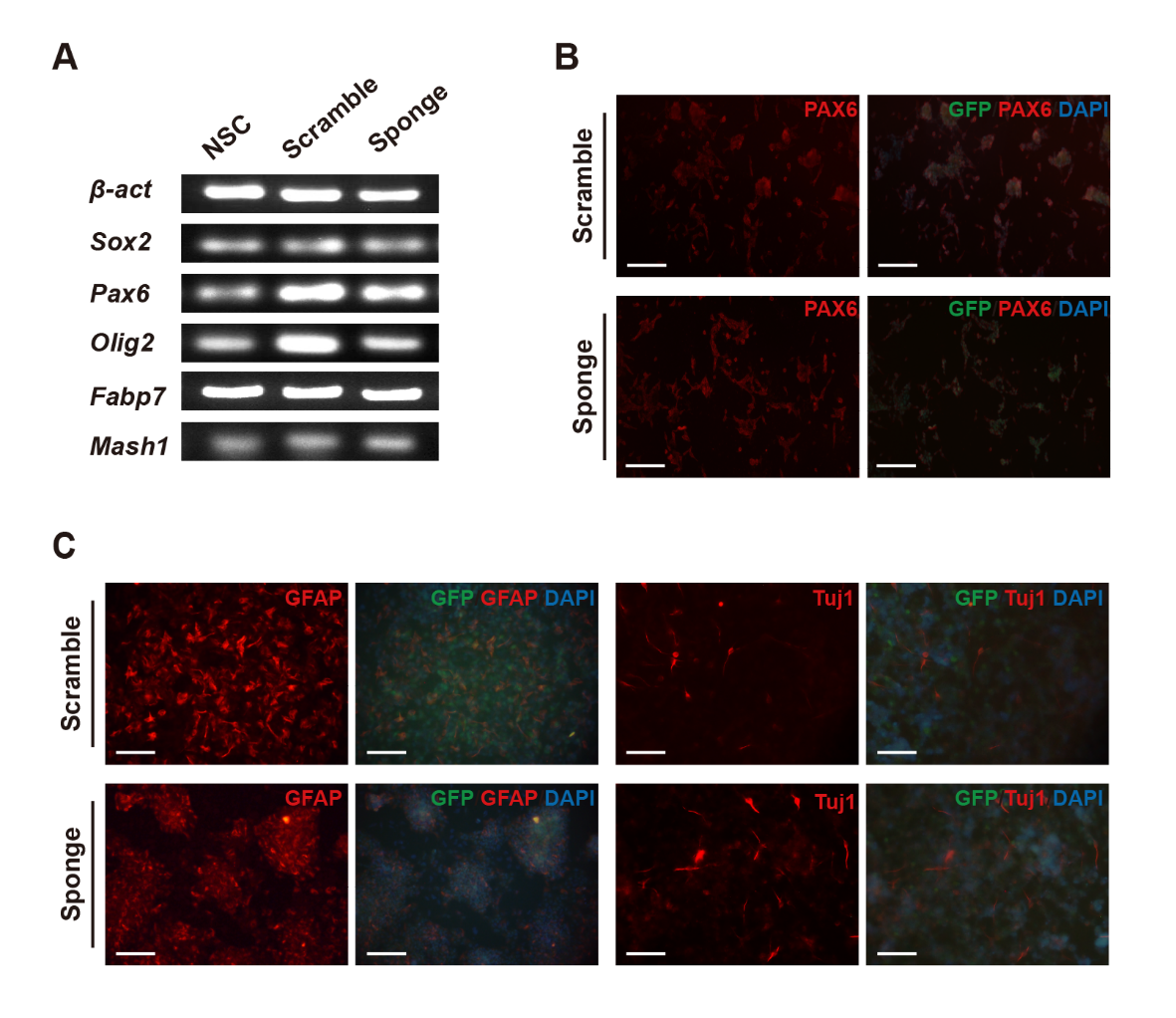


**Supplementary Figure 3.** **Establishment of GFP-Positive Infected Cells.** **(A)** Representative RT-PCR data shows mRNA expression of NSC markers (*Sox2*, *Pax6*, *Olig2*, *Fabp7*, and *Mash1*) in mock NSCs, scramble NSCs, and sponge NSCs. **(B)** Representative images showing immunofluorescence staining of PAX6 in scramble NSCs and sponge NSCs. **(C)** Representative immunofluorescence images of TUJ1 positive neurons and GFAP positive astrocytes in sponge NSCs and scramble NSCs. Scale bars: 250 μm.


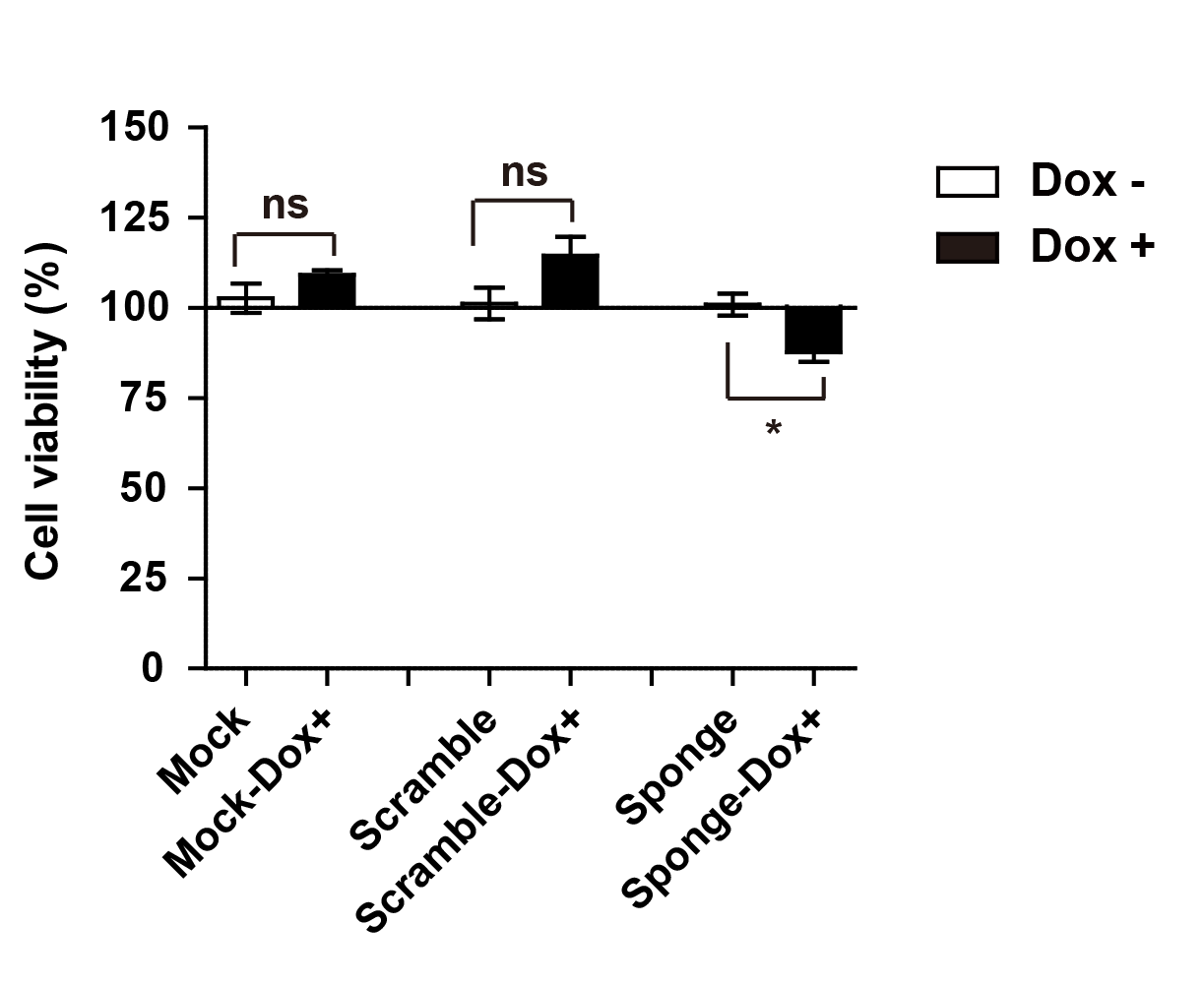


**Supplementary Figure 4.** Representative cell viability bar graphs of mock NSCs, scramble NSCs, and sponge NSCs after induction down-regulation of microRNA-325-3p. Graphs represent changes after normalization to non-doxycycline treat. Data are presented as means ± SEM (*n* = 3). **p* < 0.05.

**1.2 Supplementary Tables**

**Supplementary Table 1. Primer sequences for PCR**

| Gene | Sequences (5′-3′) | Usage |
| --- | --- | --- |
| *Sox2* | F-TTC GAG GAA AGG GTT CTT GCT | RT-PCR |
|  | R-TCC TTC GTT TGT AAC GGT CCT |  |
| *Pax6* | F-CGC GGA TCT GTG TTG CTC AT | RT-PCR |
|  | R-CTT AAA TCC ATG GCA AAT CTT GTC G |  |
| *Olig2* | F-GAC AAG AAG CAG ATG ACT GAG C | RT-PCR |
|  | R-TGG CGA TGT TGA GGT CGT |  |
| *Fabp7* | F-GGG TAA GAC CCG AGT TCC TC | RT-PCR |
|  | R-ATC ACC ACT TTG CCA CCT TC |  |
| *Mash1* | F-CTC GTC CTC TCC GGA ACT GAT G | RT-PCR |
|  | R-CGA CAG GAC GCC GCG CTG AAA G |  |
| *β-actin* | F-CGT GCG TGA CAT CAA AGA GAA GC | RT-PCR |
|  | R-ATC TGC TGG AAG GTG GAC AGT GAG |  |
| *Bai1* | F-CAG CTC ATG ACC GAC TTT GA | qRT-PCR |
|  | R-TGC AGC TTG GAC ACA TTA GC |  |
| *β-actin* | F-CGT GCG TGA CAT CAA AGA GAA GC | qRT-PCR |
| *mir-16* | F-TAG CAG CAC GTA AAT ATT GGC G | qRT-PCR |
| *mir-325-3p* | F-TTT ATT GAG CAC CTC CTA TCA A | qRT-PCR |
| *Universal* | F-GAA TCG AGC ACC AGT TAC GCA TGC CG | qRT-PCR |

F, forward primer; R, reverse primer; qRT-PCR, quantitative RT-PCR;

**Supplementary Table 2. Antibodies for immunocytochemistry**

| Name | Source | Dilution |
| --- | --- | --- |
| PAX6 | DSHB (Iwoa, IA, US) | 1:300 |
| GFAP | Dako (Carpineteria, CA, US) | 1:300 |
| TUJ1 | Millipore (Billerica, MA, US) | 1:300 |
| DAPI | Sigma-Aldrich (St. Louis, MO, US) | 1:1000 |
| Anti-mouse IgG Fab2 Alexa 488 | Cell Signaling Technologies (Danvers, MA, US) | 1:1000 |
| Anti-rabbit IgG Fab2 Alexa 555 | Cell Signaling Technologies | 1:1000 |

**Supplementary Table 3. Antibodies for western blot**

| Name | Source | Dilution |
| --- | --- | --- |
| β-ACTIN | Santa Cruz Biotechnology (Dallas, TX, US) | 1:1000 |
| BAI1 | Novus Biologicals (Centennial, CO, US) | 1:1000 |
| Anti-mouse IgG-HRP | Santa Cruz Biotechnology | 1:1000 |
| Anti-rabbit IgG-HRP | Cell Signaling Technologies | 1:1000 |
